# Anterior Sacroiliac Fracture Dislocation: A Comparative Radiologic analysis of Crescent Fractures in Pelvic Ring Injuries: A Retrospective Study

**DOI:** 10.3390/medicina60081375

**Published:** 2024-08-22

**Authors:** You-Seung Chun, Kyeong-Eon Kwon, Se-Won Lee

**Affiliations:** 1 Department of Orthopedic Surgery, Uijeongbu St. Mary’s Hospital, College of Medicine, The Catholic University of Korea, Uijeongbu 11765, Republic of Korea; icryan2002@gmail.com; 2 Department of Orthopedic Surgery, Yeouido St. Mary’s Hospital, College of Medicine, The Catholic University of Korea, Seoul 07345, Republic of Korea; brighting1213@gmail.com

**Keywords:** crescent fracture, anterior sacroiliac fracture dislocation, vertical displacement, lateral compression fracture

## Abstract

*Background and Objectives*: Anterior sacroiliac fracture dislocation (ASFD), also known as locked pelvis, is a rarely reported diagnosis. The types of ASFDs are often misdiagnosed as lateral compression fractures due to the presence of crescent fractures. In this study, we distinguished ASFD from lateral compression fractures (LC 2) and studied their characteristics. *Materials and Methods*: This is a retrospective study involving patients from a Level 1 trauma center. Fifty-nine patients under the age of 65 years with crescent fractures caused by a high-energy mechanism were investigated. *Results*: The incidence of ASFD was 25% (15 of 59) in patients with crescent fractures. Among the 15 patients, 6 had override of the ilium over the sacrum, inhibiting reduction in the sacroiliac joint. Pre-operative radiographic evaluations revealed that vertical displacement of the ASFD was larger than that of lateral compression fracture (LC 2) in the outlet view (mean 9.5 vs. 1.9 mm, *p* = 0.013), and the pelvic asymmetry ratio was larger in ASFD (mean 7.8 vs. 4.1, *p* = 0.006) in the pelvis AP view. All patients achieved union after surgery. Post-operative radiography showed no significant vertical displacement difference. There was no difference in vascular injury or hemodynamic instability requiring embolization or preperitoneal pelvic packing (PPP) between the two groups. *Conclusions*: Patients with ASFD have greater vertical displacement and asymmetry compared to patients with LC 2 fractures. These fractures must be distinguished for appropriate reduction and anterior plate fixation.

## 1. Introduction

There are a few classifications used for pelvic fracture. Young and Burgess [1] classified pelvic ring injuries according to external forces, and Tile classified pelvic ring injuries according to the stability of the pelvic ring. Among Young and Burgess classification, lateral compression injury is the most common type [2]. Crescent fractures are classified as lateral compression type 2 (LC2) injuries [3,4]. They are usually displaced into the pelvic cavity, deforming the pelvic ring. Medially directed force to the pelvic ring leads to a fracture of the iliac wing with a stable posterior crescent component. Applying outward force was necessary to reduce the fractured pelvic ring. Day et al. [4] classified crescent fractures by location of the fracture line to create a guideline for fixation construction.

However, a typical type of crescent fracture had vertical displacement rather than lateral displacement. This type of fracture exhibits a unique deformity that distinguishes itself from lateral compression injury and requires reduction force in different directions due to vertical displacement [5]. In this study, we specified this type of fracture as anterior sacroiliac fracture dislocation (ASFD). Extreme cases of this fracture were reported by some studies [5,6]. In extreme ASFD, the iliac wing overrides the sacrum superiorly and anteriorly, preventing closed reduction. This extreme type of injury is easily recognized. However, similar fracture displacement patterns can be observed in patients whose ilium does not override the sacrum. These similar fracture patterns were not classified before.

We therefore addressed the following: (1) study the characteristics of ASFD, (2) compare the displacement pattern to that of LC 2 fracture, and (3) find a radiological factor that can distinguish ASFD from crescent fracture.

## 2. Methods

**Figure 1:**
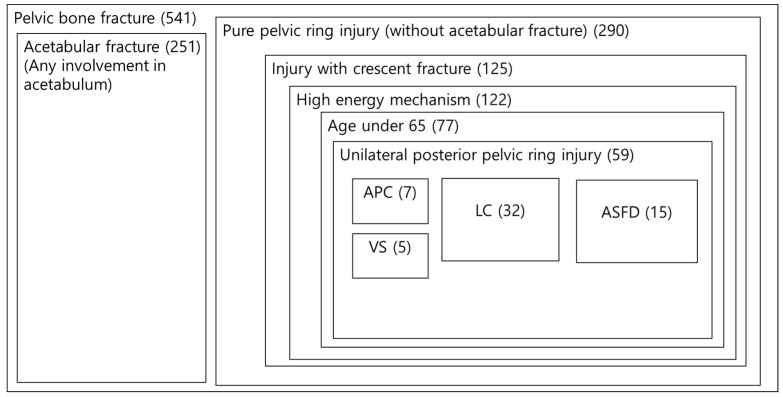
Retrospective patient classification chart. Numbers in the parentheses are the number of patients in the group. APC, anteroposterior compression, LC, lateral compression, ASFD, anterior sacroiliac fracture dislocation, and VS, vertical shear.

Patients in Level 1 trauma centers who were treated surgically for pelvic bone fractures from March 2015 to December 2023 were collected. Of the 541 total patients, 251 were excluded due to acetabular fracture, leaving 290 patients with only a pelvic ring injury. Of these, 125 patients had a crescent fracture, of whom 122 were injured by a high-energy mechanism, and 77 were younger than 65 years. Finally, appropriate unilateral posterior pelvic ring injury was analyzed in 59 patients (Figure 1). Medical records for patients were obtained to collect injury mechanisms and the history of initial treatments such as preperitoneal pelvic packing (PPP) or embolization.

### 2.1. Classification of Fracture

We classified fractures according to the Young and Burgess classification [1,3]. Patients with crescent fractures showed distinctive patterns. Anteroposterior compression injury exhibited symphysis widening that was visible on the pelvis AP view. Lateral compression injury was identified on pelvis inlet, outlet, and AP views. Typical internal rotation of the hemipelvis was identified in the pelvis inlet view. Classification of vertical shear (VS) fractures considered various characteristics including L5 transverse process fractures, sacroiliac (SI) joint diastasis, transforaminal sacral fractures, internal rotation of the hemipelvis, and diastasis of the pubic symphysis [7].

### 2.2. Definition of Anterior Sacroiliac Fracture Dislocation

**Figure 2:**
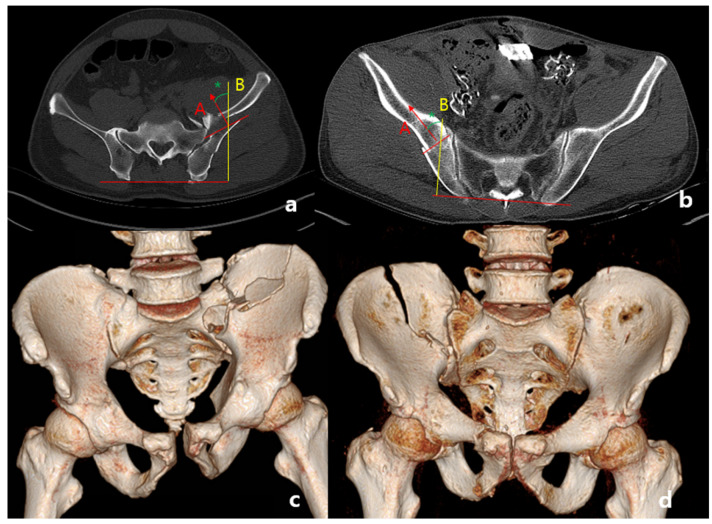
Comparing fracture lines of crescent fracture. Horizontal CT cut of ASFD (**a**) and LC (**b**). Three-dimensional reconstruction images of ASFD (**c**) and LC (**d**). * Angle between A and B is defined as crescent angle (green asterisk).

**Figure 3:**
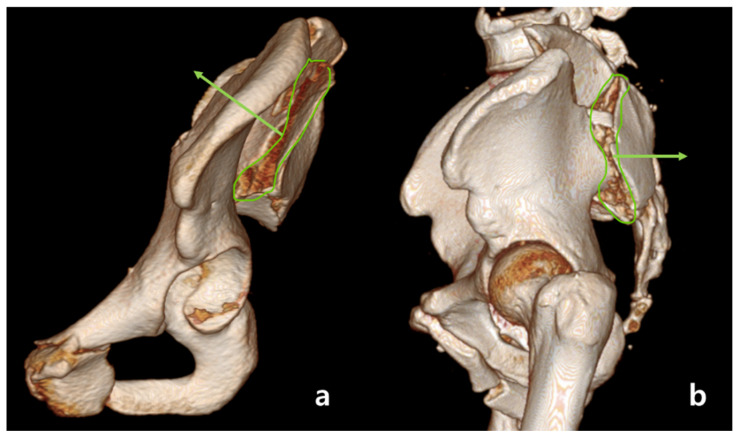
A fracture surface of the sacrum from the oblique and lateral views. The green arrows are drawn perpendicular to the fracture plane. (**a**) The sacral fracture line of ASFD appears to be directed toward the inside of the pelvic cavity. (**b**) The sacral fracture line of LC appears to be directed toward the outside of the pelvic cavity.

An anterior sacroiliac fracture dislocation (ASFD) was not defined in the Young and Burgess classification. Here, we classified ASFDs using computed tomography (CT) images. In horizontal cuts of CT images, fracture lines of crescent fractures were identified, and those facing into the pelvic cavity were classified as ASFDs. In a typical LC fracture, the fracture line of the crescent fragment points away from the pelvic cavity and is classified into three types by Day et al. [4]. For our categorization, a line perpendicular to the fracture line (A) is drawn, and the angle between this and a line (B) which is perpendicular to the line of the posterior iliac crest is measured (Figure 2). Line (A) is directed to the fractured side, not to the cortical side. Therefore, line (A) points in the direction of the fractured bone. If A is facing into the pelvic cavity, the value of the angle is positive; otherwise, it is negative (Figure 3). A patient whose angle is larger than negative 10 degrees is classified as ASFD. We defined this angle as the crescent angle. For consistency, we analyzed the largest crescent angle facing inward toward the pelvis among horizontal cuts of CT images. And for patients with fracture comminution, the fracture line with the greatest displacement was selected for measurement.

### 2.3. Radiologic Assessment

Pre-operative and post-operative pelvis AP, inlet, outlet, and oblique views were evaluated. If the patient was stabilized with an external fixator before radiography, displacement was measured after external fixation. Computed tomography was obtained for every patient. A helical CT scanner (Somatom Foce; Siemens, Erlangen, Germany) and a Picture Archiving and Communication System viewer (Marosis, Infinite, Seoul, Republic of Korea) were used with a slice thickness of 2 mm for axial, sagittal, and coronal images. The axes of the CT cuts were taken perpendicular to each other.

**Figure 4:**
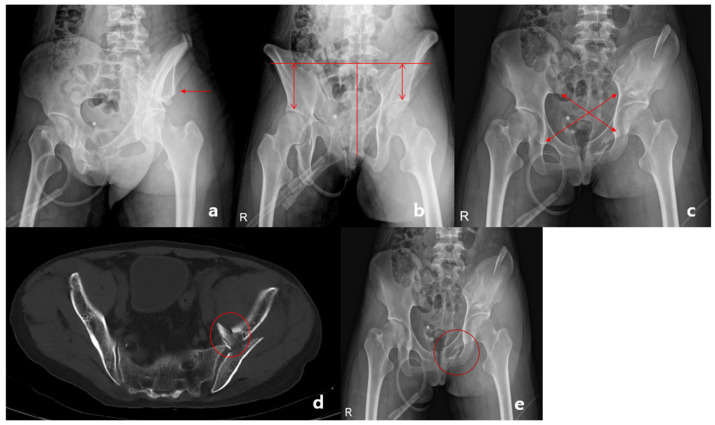
Measurement of radiological features. (**a**) Pseudo spur sign, (**b**) vertical displacement, (**c**) asymmetry ratio, and (**d**) keystone fragment (**e**) wink sign.

Oblique views of the pelvis were evaluated for pseudo spur signs and AP views were evaluated for wink signs [8] (Figure 4e). The pseudo spur sign is similar to the pathological spur sign found in associated column fractures, in which medialization of the acetabulum causes the pseudo spur sign (Figure 4a).

If a comminuted fracture was observed in the ilium, it was considered a keystone fragment. CT images were evaluated for keystone fragments of the pelvic brim and L5 transverse process fractures (Figure 4d).

### 2.4. Assessment of Vertical Displacement

The outlet view of the pelvis was used to measure vertical displacement as the distance from the acetabular roof to the superior aspect of the sacrum [9,10] (Figure 4b). To minimize measurement errors, the obliquity of the radiographs was considered. A vertical line bisecting the sacrum was drawn, and the angle between the superior aspect of the sacrum was assessed. If this angle was not perpendicular, the radiograph was deemed inadequate and excluded. Eight radiographs were excluded as they were inappropriate for analysis.

### 2.5. Assessment of Rotational Displacement

The AP view was used to measure the asymmetry of the pelvis. The distance from the inferior aspect of the sacroiliac joint to the inferior aspect of the contralateral teardrop on both sides of the pelvis was measured. The measurements were evaluated in two steps, with the first being to subtract one side from the other, yielding a pelvic asymmetry value (X − Y). The asymmetry ratio, which considers the obliquity of exposure, was then calculated as (X − Y)/(X + Y) [11,12,13] (Figure 4c).

Two orthopedic surgeons (YSC and SWL) measured each of the values independently according to guidelines. Intraclass correlation coefficients (ICCs) were calculated for the two independent measurements.

### 2.6. Statistical Analysis

The normality of data distribution for vertical displacement and the asymmetry ratio were assessed, and the scores were compared using unpaired *t*-tests. Inappropriately taken radiographs were excluded from the analysis; we had to use unpaired *t*-tests. Statistical analyses were performed using SPSS (version 21; IBM Corp., Armonk, NY, USA). The level of significance was set at *p* < 0.05.

## 3. Results

In our study population, 15 patients (25%) were classified as having an ASFD. Table 1 shows the radiological characteristics of patients with ASDFs compared to those with LC 2 fractures. There was no difference in vascular injury or hemodynamic instability requiring embolization or preperitoneal pelvic packing (PPP) between the two groups.

Pre-operative radiographic evaluation revealed greater vertical displacement with ASFDs compared to lateral compression fractures (LC 2) on the outlet view (mean 9.5 vs. 1.9 mm, *p* = 0.013), while the pelvic asymmetry ratio was larger in ASFDs (mean 7.8 vs. 4.1, *p* = 0.006) on the pelvis AP view. Post-operative radiography demonstrated no difference in vertical displacement and asymmetry (Table 2).

Day classification 1 was the majority in ASFDs, while Day classification 3 was the majority in LC 2 fractures (Table 3).

The majority of patients with ASFD were classified as Day 1 but 20 percent of patients were classified as Day 2 or 3 (Table 3).

The interclass correlation coefficient was higher than 0.8 for every measurement (Table 4).

**Figure 5:**
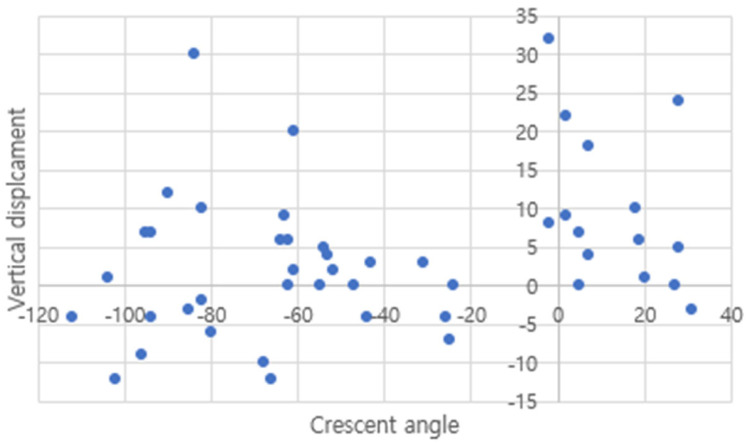
Distribution chart of patients. X-axis represents crescent angle, and Y-axis represents vertical displacement.

The crescent angle ranged from −112 degrees to 31 degrees, and vertical displacement ranged from −12 mm to 32 mm (Figure 5). The distribution chart shows the relationship between crescent angle and vertical displacement.

## 4. Discussion

Day et al. [4,14] claimed that some vertical displacement occurs in the lateral compression fracture group but seems to be limited by the sacrospinous and sacrotuberous ligaments. However, they were unable to classify which fracture type causes the most vertical displacement. In our study, the Crescent angle was defined, and crescent fractures were classified accordingly. Vertical displacement was significantly different between the ASFD and LC 2 groups. For some patients with ASFDs, the ilium was displaced over the sacrum, preventing a closed reduction.

Because an ASFD is not a previously classified fracture type, we had to set a threshold for ASFDs. There was a discontinuity in the distribution of the crescent angle in the region of −20 to 0 degrees (Figure 5). Therefore, considering measurement error, the threshold crescent angle was set to −10 degrees. The smallest crescent angle measured in ASFD patients was −2 degrees in two patients. The vertical displacement of the patient ranged from −12 mm to 32 mm. Some patients with negative crescent angles had high vertical displacement (Figure 5).

Henderson [9,15] used the AP view of the pelvis to measure vertical displacement. Matta and Tornetta [9,10] measured vertical displacement as the difference in height of the femoral heads (in Tile C1–1 and C1–2 injuries) to the superior aspect of the sacrum. Dickson and Matta [16] expressed vertical displacement as leg length discrepancy and sitting discrepancy. In this present study, we measured vertical displacement from a reference line drawn from the superior aspect of the sacrum. A vertical line bisecting the sacrum was drawn, and the two lines were verified to be perpendicular. Radiographs for the measurements were carefully selected to ensure proper visualization. In this way, high ICCs of 0.913 and 0.842 for vertical displacement were achieved. Boontanapibul et al. [17] claimed that pelvic outlet radiographs provide measurements of vertical displacement with 2 times that of the actual displacement. According to the measurements obtained in this study, patients with ASFDs appear to have a true vertical displacement of approximately 5 mm. Some recent studies focus on the vertical instability of lateral compression fractures [18,19,20]. The methods of measurement vary in these studies, but they have pointed out that some vertical displacement occurs in LC 2 fractures.

The pseudo spur sign, a distinctive radiological characteristic of ASFDs, manifests as a triangular fragment of the iliac wing visible on an obturator oblique view of the pelvis. This spur is exposed when the fractured acetabular columns are medially displaced [21]. In ASFD patients, the fractured iliac bone tends to displace both medially and superiorly, thereby revealing the pseudo spur sign. The wink sign, which stands for rotated hemipelvis, can also be seen in patients with ASFDs. The hemipelvis in LC 2 fractures tends to rotate internally, whereas the hemipelvis in ASFDs tends to rotate externally [8]. For this reason, more wink sign was observed in patients with ASFDs.

**Figure 6:**
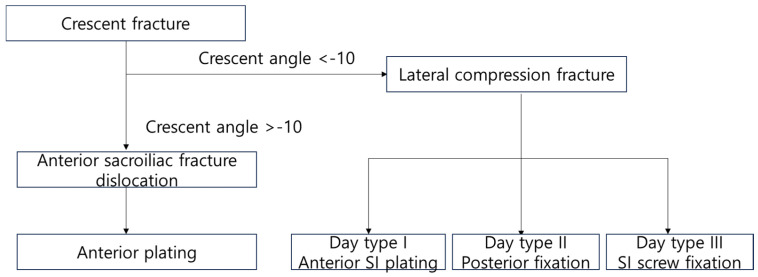
Algorithm for treatment and classification of crescent fracture.

Patients diagnosed with ASFDs underwent treatment using anterior pelvic plating, which was approached through a lateral window. Those with LC 2 fractures received treatment with either sacroiliac screws or anterior pelvic plating, depending on the fracture morphology. Previously, Day classification was used to determine the approach and fixation construct to treat patients with crescent fractures. But Day classification is not useful for patients with ASFDs, since patients with ASFDs require an anterior approach to reduce vertical displacement and need anterior plating to maintain the reduction. The patients with ASFDs and Day type III also required anterior plating, not sacroiliac screw fixation. Among our patients, 20 percent of ASFDs were classified as Day 2 or 3. Therefore, patients with crescent fractures should first be classified according to the crescent angle and, if not an ASFD, by Day classification (Figure 6). In this way, we could manage to reduce the vertical displacement of ASFD effectively, and all patients achieved bone union without vertical displacement.

Several limitations should be acknowledged. First, there may be measurement errors. To reduce measurement errors, two senior orthopedic surgeons selected and evaluated the radiographs and achieved ICC values greater than 0.8, indicating near-perfect agreement. However, the measurements required careful radiograph selection. The radiographs were selected based on strict criteria as mentioned above. Second, the crescent angle was assessed in the horizontal plane and did not fully reflect the three-dimensional shape of the fracture. The morphology and curvature of the sacrum vary among patients, resulting in the plane of horizontal cut of the CT scan not being consistent with each other. This limitation may be overcome if we acquire the plane horizontal cut parallel to the S1 upper endplate. Third, our results show that a positive crescent angle is correlated with vertical displacement, but some patients had high vertical displacement with a negative crescent angle. The crescent angle alone cannot accurately predict vertical displacement.

## 5. Conclusions

To achieve an anatomical reduction in the pelvic ring, ASFDs must be recognized, and vertical displacement and asymmetry of the pelvic ring must be considered when reducing fractures. An ASFD can be distinguished from lateral compression fracture type II by measuring the crescent angle. ASFDs have more vertical displacement and more asymmetry than LC II fractures. Anterior approach and anterior plate fixation are required for patients with ASFDs regardless of Day classification.

## Figures and Tables

**Table 1:** Radiological characteristics of fracture comparing LC fracture to ASFD.

	Lateral Compression Fracture (*n* = 32)	Anterior Sacroiliac Fracture Dislocation (*n* = 15)
Pseudo spur sign	1 (3)	14 (93)
Override of ilium on sacrum	0	6 (40)
Keystone fragment	3 (9)	9 (60)
Wink sign	2 (6)	13 (87)
Vessel injury treated with embolization	3 (9)	1 (7)
Preperitoneal pelvic packing	10 (31)	4 (27)

Numbers in parentheses are percentages.

**Table 2:** Comparison of radiological outcomes between lateral compression fracture and ASFD.

	Lateral Compression Fracture	Anterior Sacroiliac Fracture Dislocation	
	Mean ± SD	Mean ± SD	*p*-value
Pre-operative vertical displacement	1.9 ± 9.0 mm	9.5 ± 10.1 mm	0.013 ^†^
Post-operative vertical displacement	−0.5 ± 5.9 mm	4.7 ± 12.0 mm	0.061
Pre-operative asymmetry ratio (X − Y)/(X + Y)	4.1 ± 3.2%	7.8 ± 5.3%	0.006 ^†^
Post-operative asymmetry ratio	3.8 ± 2.9%	4.5 ± 3.1%	0.462

^†^ Statistically significant.

**Table 3:** Day classification for each type.

	Lateral Compression 2 (*n* = 32)	Anterior Sacroiliac Fracture Dislocation (*n* = 15)
Day 1	3 (9)	12 (80)
Day 2	7 (21)	2 (13)
Day 3	22 (68)	1 (7)

Numbers in parentheses are percentages. All patients achieved bone union post-operatively 1 year later.

**Table 4:** Intraclass correlation coefficient for measured values.

	ICC (95% CI)	*p*-Value
Angle of crescent fracture line	0.989 (0.980–0.994)	<0.001
Pre-operative vertical displacement	0.913 (0.847–0.951)	<0.001
Post-operative vertical displacement	0.842 (0.732–0.906)	<0.001
Pre-operative asymmetry ratio	0.917 (0.857–0.952)	<0.001
Post-operative asymmetry ratio	0.893 (0.815–0.939)	<0.001

ICC—Intraclass Correlation Coefficient.

## Data Availability

Previous studies included in this study can be found in PubMed.

## References

[1] Young J.W., Burgess A.R., Brumback R.J., Poka A. (1986). Pelvic fractures: Value of plain radiography in early assessment and management. Radiology.

[2] Tile M. (1988). Pelvic ring fractures: Should they be fixed?. J. Bone Jt. Surg. Br..

[3] Alton T.B., Gee A.O. (2014). Classifications in brief: Young and burgess classification of pelvic ring injuries. Clin. Orthop. Relat. Res..

[4] Day A.C., Kinmont C., Bircher M.D., Kumar S. (2007). Crescent fracture-dislocation of the sacroiliac joint: A functional classification. J. Bone Jt. Surg. Br..

[5] Trikha V., Singh V., Kumar V.S. (2015). Anterior fracture dislocation of sacroiliac joint: A rare type of crescent fracture. Indian J. Orthop..

[6] Xiao J., Wang Y., Zhang M., Jiang R., Zhu T., Liu G., Zuo J. (2017). Anterior fracture dislocation of the sacroiliac joint: A case report and literature review. Technol. Health Care.

[7] Blum L., Hake M.E., Charles R., Conlan T., Rojas D., Martin M.T., Mauffrey C. (2018). Vertical shear pelvic injury: Evaluation, management, and fixation strategies. Int. Orthop..

[8] Lee S.W., Kim W.Y., Koh S.J., Kim Y.Y. (2017). Posterior locked lateral compression injury of the pelvis in geriatric patients: An infrequent and specific variant of the fragility fracture of pelvis. Arch. Orthop. Trauma Surg..

[9] Tornetta P., Matta J.M. (1996). Outcome of operatively treated unstable posterior pelvic ring disruptions. Clin. Orthop. Relat. Res..

[10] Matta J.M., Tornetta P. (1996). Internal fixation of unstable pelvic ring injuries. Clin. Orthop. Relat. Res..

[11] Keshishyan R.A., Rozinov V.M., Malakhov O.A., Kuznetsov L.E., Strunin E.G., Chogovadze G.A., Tsukanov V.E. (1995). Pelvic polyfractures in children. Radiographic diagnosis and treatment. Clin. Orthop. Relat. Res..

[12] Bowerman J.W., Sena J.M., Chang R. (1982). The teardrop shadow of the pelvis; anatomy and clinical significance. Radiology.

[13] Lefaivre K.A., Starr A.J., Barker B.P., Overturf S., Reinert C.M. (2009). Early experience with reduction of displaced disruption of the pelvic ring using a pelvic reduction frame. J. Bone Jt. Surg. Br..

[14] Burgess A.R., Eastridge B.J., Young J.W., Ellison T.S., Ellison P.S., Poka A., Bathon G.H., Brumback R.J. (1990). Pelvic ring disruptions: Effective classification system and treatment protocols. J. Trauma Acute Care Surg..

[15] Henderson R.C. (1989). The long-term results of nonoperatively treated major pelvic disruptions. J. Orthop. Trauma.

[16] Dickson K.F., Matta J.M. (2009). Skeletal deformity after anterior external fixation of the pelvis. J. Orthop. Trauma.

[17] Boontanapibul K., Harnroongroj T., Sudjai N., Harnroongroj T. (2015). Vertical pelvic ring displacement in pelvic ring injury: Measurements in pelvic outlet radiograph and in cadavers. Indian J. Orthop..

[18] Weaver M.J., Bruinsma W., Toney E., Dafford E., Vrahas M.S. (2012). What Are the Patterns of Injury and Displacement Seen in Lateral Compression Pelvic Fractures?. Clin. Orthop. Relat. Res..

[19] Wu Y., Chen H., Zhou X., Tang P. (2022). Lateral Compression Type 2 Pelvic Fractures-A Clinical Study of Fracture Displacement Measurement and Closed Reduction. Orthop. Surg..

[20] Xiang G., Dong X., Jiang X., Cai L., Wang J., Guo X., Xiao J., Feng Y. (2021). Comparison of percutaneous cross screw fixation versus open reduction and internal fixation for pelvic Day type II crescent fracture-dislocation: Case-control study. J. Orthop. Surg. Res..

[21] Johnson T.S. (2005). The spur sign. Radiology.

